# Bis(tetra­butyl­ammonium) tetra­chlorido­manganate(II) di­chloro­methane disolvate

**DOI:** 10.1107/S2414314623006107

**Published:** 2023-07-14

**Authors:** Michael T. Hay, Hemant P. Yennawar

**Affiliations:** a Penn State Beaver, 100 University Drive, Monaca, PA 15061, USA; b The Pennsylvania State University, Dept., Biochemistry and Molecular Biology, University Park, PA 16802, USA; Vienna University of Technology, Austria

**Keywords:** crystal structure, organic–inorganic salt, manganese(II) complex, tetra­butyl­ammonium salt, solvate

## Abstract

The title compound, (C_16_H_36_N)_2_[MnCl_4_]·2CH_2_Cl_2_, is an ionic organic–inorganic hybride compound consisting of a tetra­butyl­ammonium cation and a tetra­chlorido­manganate(II) anion in a 2:1 stoichiometric ratio. The cation contains a central nitro­gen atom bonded to four *n*-butyl groups in a tetra­hedral arrangement, while the anion contains a central Mn^II^ atom tetra­hedrally coordinated by four chlorido ligands. It co-crystallized with two equivalents of di­chloro­methane solvent, CH_2_Cl_2_, to give the following empirical formula: [(C_4_H_9_)_4_N]_2_[MnCl_4_]·(CH_2_Cl_2_)_2_. The crystal structure is mainly stabilized by Coulombic inter­actions.

## Abstract 2

The ionic title compound consists of a 2:1 ratio of the tetra­butyl­ammonium cation (1+) and the tetra­chlorido­manganate(II) anion (2–). The structure reported contains two di­chloro­methane solvent mol­ecules co-crystallized per anion.

## Graphical abstract

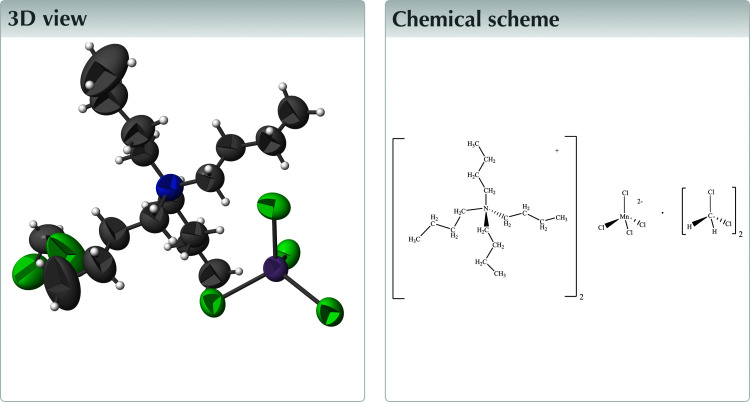

## Structure description

During our efforts to prepare novel manganese-containing coordination complexes, we synthesized the previously reported non-solvated compound bis­(tetra­butyl­ammonium) tetra­chlorido­manganate(II). In conducting our experiments, we inadvertently obtained the disolvated title compound and determined its crystal structure. After reviewing the literature, we realised that no crystallographic data had yet been reported on either the non-solvated or solvated forms of this substance. The only crystallographic data related to this system was the powder X-ray diffraction data for the non-solvated form at 900 K after it had already undergone thermal decomposition (Styczeń *et al.*, 2009 ▸). Herein we present the results of the single-crystal structure analysis of the title compound.

**Figure 1:**
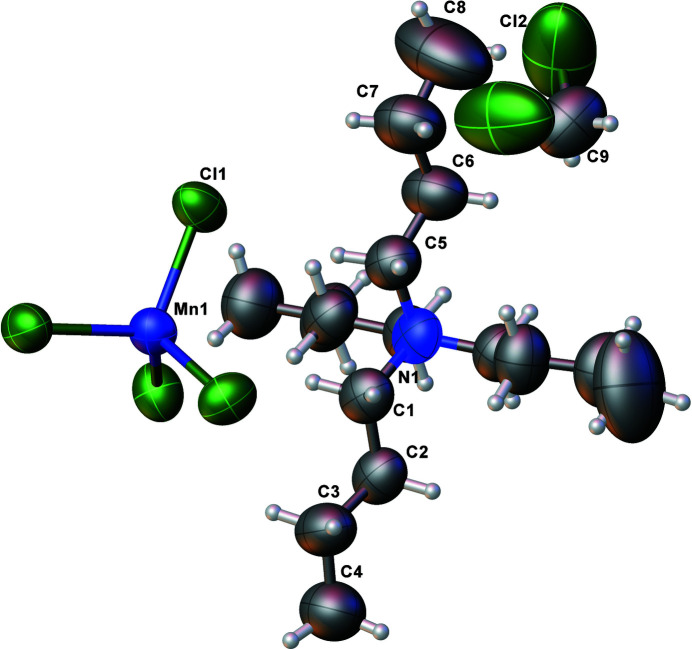
Mol­ecular structures of the entities present in the title compound, with displacement ellipsoids drawn at the 50% probability level.

The structural formula shows a ratio of 2:1 for the tetra­butyl­ammonium cation and the tetra­chlorido­manganate(II) anion, combined with two solvent mol­ecules of di­chloro­methane (Fig. 1 ▸). The above three mol­ecular entities have inter­nal symmetries allowing them to occupy different special positions in the lattice with point group symmetries 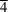 .. (multiplicity 4, Wyckoff letter *a*) for the anion, and .2. (8 *d*) both for the cation and the solvent mol­ecule. The root-mean-square deviations from ideal *T_d_* symmetry for the anion, *S*_4_ symmetry for the cation and *C*_2*v*_ symmetry for the solvent mol­ecule amount to 0.0123, 0.0501 and 0 Å, respectively, as calculated with *PLATON* (Spek, 2020 ▸), based on the *SYMMOL* program by Pilati & Forni (1998 ▸, 2000 ▸). The tetra­butyl­ammonium cation, (C_4_H_9_)_4_N^+^, consists of a central nitro­gen atom tetra­hedrally surrounded by ordered butyl groups, with N—C bond lengths ranging from 1.505 (12) Å to 1.511 (11) Å and C—N—C bond angles in the range of 105.8 (5)–111.7 (11)°. The complex anion MnCl_4_^2–^ is consistent with the structure previously published for the tetra­methyl­ammonium salt (Rodríguez-Lazcano *et al.*, 2009 ▸) – the central Mn^II^ atom is bound with four chloride ligands tetra­hedrally arranged. The Cl—Mn—Cl bond angles are 108.80 (12)-109.81 (12)°. The Mn—Cl bond lengths are all 2.364 (2) Å.

**Figure 2:**
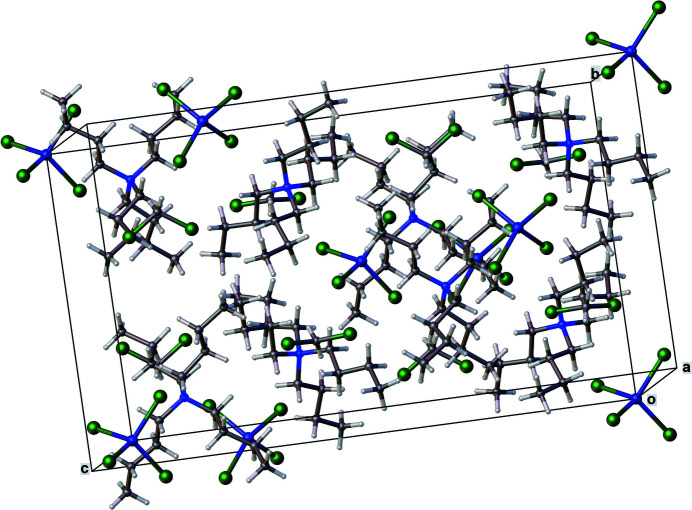
Packing diagram of the crystal structure, which is stabilized primarily by Coulombic forces.

The crystal structure (Fig. 2 ▸) is stabilized primarily by Coulombic forces in the absence of classical hydrogen-bonding inter­actions.

## Synthesis and crystallization

A similar protocol was followed as previously reported in the literature (Styczeń *et al.*, 2009 ▸). Pink MnCl_4_·4H_2_O (5.05 mmol, 1.00 g) was dissolved in warm absolute ethanol (10–15 ml). Separately, two equivalents of white (C_4_H_9_)_4_NCl·H_2_O (10.1 mmol, 2.81 g) were also dissolved in warm absolute ethanol (10–15 ml). The two ethanol solutions were then mixed, and the solution turned a light-green color. The ethanol was removed under reduced pressure with heating to produce a pale-green solid. The solid was recrystallized from di­chloro­methane/ether to give pale-green crystals. After drying the crystals under reduced pressure at 311 K, they were massed (3.07 g, 89.2% yield). They were analyzed by IR and elemental analysis. IR (cm^−1^): 2962*m*, 2943*m*, 2875*m*, 1484*s*, 1468*m*, 1378*m*, 1151*w*, 1025*w*, 881*m*, 749*m*, 732*m*. Analysis calculated for (C_16_H_36_N)_2_MnCl_4_: C, 56.38; H, 10.65, N, 4.11. Found: C, 56.47; H, 11.47, N, 4.04. X-ray quality crystals were obtained from a mixture of di­chloro­methane/ether during a reaction involving the non-solvated form of the title compound as the starting material.

## Refinement

Crystal data, data collection and structure refinement details for the reported structure is summarized in Table 1 ▸. The crystal diffracted poorly at high resolution. The average intensity drops below the 3σ level at 0.9933 Å. Consequently, the reliability factors are comparatively high. As a result of the special symmetry of the di­chloro­methane solvent mol­ecule, the two H atoms (H9*A* and H9*B*) were refined with half-occupancy.

## Supplementary Material

Crystal structure: contains datablock(s) I. DOI: 10.1107/S2414314623006107/wm4191sup1.cif

Structure factors: contains datablock(s) I. DOI: 10.1107/S2414314623006107/wm4191Isup2.hkl

CCDC reference: 2280618

Additional supporting information:  crystallographic information; 3D view; checkCIF report

## Figures and Tables

**Table 1:** Experimental details

Crystal data	
Chemical formula	(C_16_H_36_N)_2_[MnCl_4_]·2CH_2_Cl_2_
*M*_r_	851.50
Crystal system, space group	Tetragonal, *I* 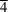 2*d*
Temperature (K)	173
*a*, *c* (Å)	14.0775 (3), 24.3492 (8)
*V* (Å^3^)	4825.4 (3)
*Z*	4
Radiation type	Cu *K*α
μ (mm^−1^)	6.46
Crystal size (mm)	0.38 × 0.28 × 0.13

Data collection	
Diffractometer	ROD, Synergy Custom system, HyPix-Arc 150
Absorption correction	Analytical (*CrysAlis PRO*; Rigaku OD, 2021 ▸)
*T*_min_, *T*_max_	0.060, 0.359
No. of measured, independent and observed [*I* > 2σ(*I*)] reflections	9265, 2334, 1567
*R*_int_	0.038
(sin θ/λ)_max_ (Å^−1^)	0.624

Refinement	
*R*[*F* ^2^ > 2σ(*F* ^2^)], *wR*(*F* ^2^), *S*	0.075, 0.222, 1.06
No. of reflections	2334
No. of parameters	108
No. of restraints	47
H-atom treatment	H atoms treated by a mixture of independent and constrained refinement
Δρ_max_, Δρ_min_ (e Å^−3^)	0.35, −0.32
Absolute structure	Flack *x* determined using 458 quotients [(*I* ^+^)-(*I* ^-^)]/[(*I* ^+^)+(*I* ^-^)] (Parsons *et al.*, 2013 ▸)
Absolute structure parameter	−0.018 (8)

Computer programs: *CrysAlis PRO* (Rigaku OD, 2021 ▸), *OLEX2.solve* (Bourhis *et al.*, 2015 ▸), *SHELXL2018/3* (Sheldrick, 2015 ▸), and *OLEX2* (Dolomanov *et al.*, 2009 ▸).

## full crystallographic data

### Crystal data

**Table:**

(C_16_H_36_N)_2_[MnCl_4_]·2CH_2_Cl_2_	*D*_x_ = 1.172 Mg m^−^^3^
*M**_r_* = 851.50	Cu *K*α radiation, λ = 1.54184 Å
Tetragonal, *I*42*d*	Cell parameters from 3369 reflections
*a* = 14.0775 (3) Å	θ = 3.6–60.8°
*c* = 24.3492 (8) Å	µ = 6.46 mm^−^^1^
*V* = 4825.4 (3) Å^3^	*T* = 173 K
*Z* = 4	Plate, clear yellow
*F*(000) = 1820	0.38 × 0.28 × 0.13 mm

### Data collection

**Table:**

ROD, Synergy Custom system, HyPix-Arc 150 diffractometer	2334 independent reflections
Radiation source: Rotating-anode X-ray tube, Rigaku (Cu) X-ray Source	1567 reflections with *I* > 2σ(*I*)
Mirror monochromator	*R*_int_ = 0.038
Detector resolution: 10.0000 pixels mm^-1^	θ_max_ = 74.1°, θ_min_ = 3.6°
ω scans	*h* = −17→16
Absorption correction: analytical (*CrysAlisPro*; Rigaku OD, 2021)	*k* = −16→17
*T*_min_ = 0.060, *T*_max_ = 0.359	*l* = −27→29
9265 measured reflections	

### Refinement

**Table:**

Refinement on *F*^2^	Hydrogen site location: mixed
Least-squares matrix: full	H atoms treated by a mixture of independent and constrained refinement
*R*[*F*^2^ > 2σ(*F*^2^)] = 0.075	*w* = 1/[σ^2^(*F*_o_^2^) + (0.1133*P*)^2^ + 4.1001*P*] where *P* = (*F*_o_^2^ + 2*F*_c_^2^)/3
*wR*(*F*^2^) = 0.222	(Δ/σ)_max_ < 0.001
*S* = 1.06	Δρ_max_ = 0.35 e Å^−^^3^
2334 reflections	Δρ_min_ = −0.32 e Å^−^^3^
108 parameters	Absolute structure: Flack *x* determined using 458 quotients [(*I*^+^)-(*I*^-^)]/[(*I*^+^)+(*I*^-^)] (Parsons *et al.*, 2013)
47 restraints	Absolute structure parameter: −0.018 (8)
Primary atom site location: iterative	

### Special details

**Table:**

Geometry. All esds (except the esd in the dihedral angle between two l.s. planes) are estimated using the full covariance matrix. The cell esds are taken into account individually in the estimation of esds in distances, angles and torsion angles; correlations between esds in cell parameters are only used when they are defined by crystal symmetry. An approximate (isotropic) treatment of cell esds is used for estimating esds involving l.s. planes.

### Fractional atomic coordinates and isotropic or equivalent isotropic displacement parameters (Å^2^)

**Table:**

	*x*	*y*	*z*	*U*_iso_*/*U*_eq_	
Mn1	0.500000	0.000000	0.750000	0.0722 (7)	
Cl1	0.37996 (17)	−0.06513 (19)	0.69347 (9)	0.0925 (8)	
Cl2	0.2523 (6)	0.1511 (5)	0.4325 (2)	0.248 (4)	
C9	0.250000	0.2191 (18)	0.375000	0.152 (9)	
H9	0.204 (11)	0.254 (12)	0.382 (8)	0.182*	
N1	0.3681 (8)	0.250000	0.625000	0.090 (3)	
C1	0.4291 (7)	0.2541 (7)	0.6760 (3)	0.094 (3)	
H1A	0.466853	0.194827	0.677948	0.112*	
H1B	0.386692	0.255178	0.708408	0.112*	
C2	0.4966 (8)	0.3374 (6)	0.6802 (3)	0.100 (3)	
H2A	0.540087	0.337104	0.648222	0.120*	
H2B	0.459916	0.397367	0.679322	0.120*	
C3	0.5535 (9)	0.3328 (8)	0.7322 (5)	0.125 (4)	
H3A	0.587481	0.271263	0.733181	0.150*	
H3B	0.509107	0.334000	0.763696	0.150*	
C4	0.6252 (10)	0.4116 (9)	0.7396 (6)	0.142 (5)	
H4A	0.664940	0.416247	0.706657	0.213*	
H4B	0.665249	0.397709	0.771501	0.213*	
H4C	0.591855	0.471854	0.745395	0.213*	
C5	0.3081 (8)	0.1621 (7)	0.6308 (4)	0.101 (3)	
H5A	0.270075	0.167990	0.664871	0.121*	
H5B	0.350980	0.106950	0.635531	0.121*	
C6	0.2413 (9)	0.1411 (9)	0.5839 (5)	0.127 (4)	
H6A	0.278449	0.128709	0.550114	0.152*	
H6B	0.200173	0.196905	0.577132	0.152*	
C7	0.1799 (11)	0.0552 (10)	0.5969 (6)	0.155 (5)	
H7A	0.221001	0.001495	0.608109	0.186*	
H7B	0.137194	0.070402	0.627982	0.186*	
C8	0.1215 (16)	0.026 (2)	0.5479 (8)	0.260 (13)	
H8A	0.162622	0.022088	0.515483	0.390*	
H8B	0.071993	0.074082	0.541378	0.390*	
H8C	0.091990	−0.035380	0.554830	0.390*	

### Atomic displacement parameters (Å^2^)

**Table:**

	*U*^11^	*U*^22^	*U*^33^	*U*^12^	*U*^13^	*U*^23^
Mn1	0.0805 (10)	0.0805 (10)	0.0555 (13)	0.000	0.000	0.000
Cl1	0.0873 (14)	0.1130 (18)	0.0771 (12)	0.0069 (13)	−0.0096 (11)	−0.0207 (12)
Cl2	0.359 (8)	0.258 (7)	0.127 (3)	−0.111 (7)	−0.053 (5)	0.044 (4)
C9	0.17 (2)	0.137 (19)	0.145 (18)	0.000	0.050 (17)	0.000
N1	0.115 (8)	0.085 (7)	0.070 (6)	0.000	0.000	0.020 (5)
C1	0.122 (7)	0.096 (6)	0.063 (5)	−0.006 (6)	0.003 (5)	0.014 (4)
C2	0.130 (7)	0.093 (6)	0.077 (5)	0.000 (7)	0.003 (6)	0.009 (5)
C3	0.152 (10)	0.106 (8)	0.117 (9)	−0.022 (8)	−0.028 (8)	0.008 (6)
C4	0.146 (10)	0.140 (11)	0.139 (11)	−0.018 (9)	−0.018 (9)	0.005 (9)
C5	0.117 (8)	0.098 (7)	0.088 (6)	−0.007 (6)	0.000 (6)	0.008 (5)
C6	0.141 (10)	0.131 (10)	0.109 (8)	−0.030 (9)	−0.017 (8)	0.008 (7)
C7	0.166 (13)	0.163 (12)	0.136 (11)	−0.029 (11)	−0.028 (10)	−0.005 (10)
C8	0.22 (2)	0.32 (3)	0.24 (2)	−0.11 (2)	−0.07 (2)	0.06 (2)

### Geometric parameters (Å, º)

**Table:**

Mn1—Cl1^i^	2.364 (2)	C3—H3B	0.9900
Mn1—Cl1^ii^	2.364 (2)	C3—C4	1.510 (10)
Mn1—Cl1^iii^	2.364 (2)	C4—H4A	0.9800
Mn1—Cl1	2.364 (2)	C4—H4B	0.9800
Cl2—C9	1.695 (15)	C4—H4C	0.9800
C9—H9	0.83 (15)	C5—H5A	0.9900
C9—H9^iv^	0.83 (15)	C5—H5B	0.9900
N1—C1^v^	1.511 (11)	C5—C6	1.510 (10)
N1—C1	1.511 (11)	C6—H6A	0.9900
N1—C5	1.505 (12)	C6—H6B	0.9900
N1—C5^v^	1.505 (12)	C6—C7	1.519 (11)
C1—H1A	0.9900	C7—H7A	0.9900
C1—H1B	0.9900	C7—H7B	0.9900
C1—C2	1.513 (9)	C7—C8	1.505 (11)
C2—H2A	0.9900	C8—H8A	0.9800
C2—H2B	0.9900	C8—H8B	0.9800
C2—C3	1.500 (9)	C8—H8C	0.9800
C3—H3A	0.9900		
			
Cl1^i^—Mn1—Cl1^ii^	109.81 (6)	H3A—C3—H3B	107.5
Cl1^ii^—Mn1—Cl1	108.80 (12)	C4—C3—H3A	108.5
Cl1^i^—Mn1—Cl1^iii^	108.80 (12)	C4—C3—H3B	108.5
Cl1^iii^—Mn1—Cl1	109.81 (6)	C3—C4—H4A	109.5
Cl1^i^—Mn1—Cl1	109.81 (6)	C3—C4—H4B	109.5
Cl1^ii^—Mn1—Cl1^iii^	109.81 (6)	C3—C4—H4C	109.5
Cl2—C9—Cl2^iv^	111.3 (14)	H4A—C4—H4B	109.5
Cl2^iv^—C9—H9	120 (10)	H4A—C4—H4C	109.5
Cl2—C9—H9	100 (10)	H4B—C4—H4C	109.5
Cl2^iv^—C9—H9^iv^	100 (10)	N1—C5—H5A	108.3
Cl2—C9—H9^iv^	120 (10)	N1—C5—H5B	108.3
H9—C9—H9^iv^	107 (10)	N1—C5—C6	116.1 (8)
C1—N1—C1^v^	110.7 (10)	H5A—C5—H5B	107.4
C5^v^—N1—C1^v^	105.8 (5)	C6—C5—H5A	108.3
C5—N1—C1	105.8 (5)	C6—C5—H5B	108.3
C5—N1—C1^v^	111.4 (6)	C5—C6—H6A	109.5
C5^v^—N1—C1	111.4 (6)	C5—C6—H6B	109.5
C5—N1—C5^v^	111.7 (11)	C5—C6—C7	110.6 (9)
N1—C1—H1A	108.2	H6A—C6—H6B	108.1
N1—C1—H1B	108.2	C7—C6—H6A	109.5
N1—C1—C2	116.2 (7)	C7—C6—H6B	109.5
H1A—C1—H1B	107.4	C6—C7—H7A	109.4
C2—C1—H1A	108.2	C6—C7—H7B	109.4
C2—C1—H1B	108.2	H7A—C7—H7B	108.0
C1—C2—H2A	109.4	C8—C7—C6	111.0 (12)
C1—C2—H2B	109.4	C8—C7—H7A	109.4
H2A—C2—H2B	108.0	C8—C7—H7B	109.4
C3—C2—C1	111.0 (7)	C7—C8—H8A	109.5
C3—C2—H2A	109.4	C7—C8—H8B	109.5
C3—C2—H2B	109.4	C7—C8—H8C	109.5
C2—C3—H3A	108.5	H8A—C8—H8B	109.5
C2—C3—H3B	108.5	H8A—C8—H8C	109.5
C2—C3—C4	115.2 (9)	H8B—C8—H8C	109.5
			
N1—C1—C2—C3	−179.5 (10)	C1—C2—C3—C4	178.4 (11)
N1—C5—C6—C7	−175.7 (11)	C5^v^—N1—C1—C2	−58.2 (12)
C1^v^—N1—C1—C2	59.3 (7)	C5—N1—C1—C2	−179.8 (9)
C1^v^—N1—C5—C6	−58.6 (12)	C5^v^—N1—C5—C6	59.6 (8)
C1—N1—C5—C6	−179.0 (10)	C5—C6—C7—C8	−173.2 (16)

Symmetry codes: (i) *y*+1/2, −*x*+1/2, −*z*+3/2; (ii) −*x*+1, −*y*, *z*; (iii) −*y*+1/2, *x*−1/2, −*z*+3/2; (iv) −*x*+1/2, *y*, −*z*+3/4; (v) *x*, −*y*+1/2, −*z*+5/4.
